# Retraction: Pretransplant Prediction of Posttransplant Survival for Liver Recipients with Benign End-Stage Liver Diseases: A Nonlinear Model

**DOI:** 10.1371/journal.pone.0222109

**Published:** 2019-08-29

**Authors:** The PLOS ONE Editors

Concerns have been raised that the transplants performed in the local context at the time of procedures reported in this article [1] may have involved organs/tissues procured from prisoners [2].

Details as to the donor sources were not reported in [1], and the authors did not clarify this matter or the cause(s) of donor death in response to the journal’s post-publication inquiries. The authors stated in the article [1] that none of the transplant grafts were obtained from executed prisoners or other institutionalized persons, that all organs were contributed voluntarily, and that all donors or their families provided written informed consent for donation. However, in response to journal requests the authors did not provide documentation or consent forms to support these claims. International ethical standards call for transparency in organ donor and transplantation programs and clear informed consent procedures including considerations to ensure that donors are not subject to coercion [3,4,5].

In addition, the ethics statement in the article notes that the transplant procedures were approved by the Medical Ethics Committee of West China Hospital but the authors did not report whether this specific study was reviewed and approved by a research ethics committee, and they did not provide ethics approval documentation when requested by the journal.

The authors confirmed that the underlying data and laboratory records are not available to support results reported in the article.

Owing to the lack of documentation to demonstrate that this study had prospective ethical approval, insufficient reporting, unresolved concerns around the source of transplanted organs, lack of data and supporting documentation for the study, and in compliance with international ethical standards for organ/tissue donation and transplantation, the *PLOS ONE* Editors retract this article.

The corresponding author apologized and requested withdrawal of the article when they notified the journal office of the unavailable data and laboratory records, but the authors did not respond to the retraction decision.
